# Let's not forget those who forget! Participatory design in the context of dementia built environment

**DOI:** 10.1093/eurpub/ckac129.567

**Published:** 2022-10-25

**Authors:** E Chrysikou, E Savvopoulou, J Rehn, L Minetou, E Hernandez Garcia, H Lad, S Capolongo, D Cadar, M Orlu

**Affiliations:** 1 Bartlett School of Sustainable Construction, University College London, London, UK; 2 School of Medicine, University of Crete, Crete, Greece; 3 SynThesis Architects, Athens, Greece; 4 Faculty of Design, Darmstadt University of Applied Sciences, Darmstadt, Germany; 5 University of Stirling, Stirling, UK; 6 School of Architecture, University College London, London, UK; 7 Department Architecture, Built Environment and Construction Engineering, Politecnico di Milano, Milan, Italy; 8 Brighton and Sussex Medical School, University of Sussex, Brighton, UK; 9 School of Pharmacy, University College London, London, UK

## Abstract

Dementia is a major cause of disability and dependency among older people worldwide. Eco-bio-psychosocially supportive design can significantly reduce agitation and depression while improving mobility and daily activities. For this we need to include dementia patients as experts while understanding the neurological changes and functional impairments associated with the progression of the disease over time. How can we support dementia patients to participate? What tools/processes can we use to involve them in the design process? The aim of this project was to map and evaluate co-design methods for dementia and neurodiversity, in order to create an eco-bio-psychosocially supportive environment. Mixed methods were used comprising a systematic literature review on co-design techniques for spaces for dementia, three workshops: a 3-day one with seven early career researchers translating patient involving methodologies to the dementia context, a round table Patient and Public Involvement and Engagement with six service providers and stakeholders cross three countries and a cross-sectoral international day conference with four academics, four early career researchers and eight stakeholders and a series of co-design workshops for dementia and neurodiversity, which were then classified according to applicability so as to generate co-production methods for living environments for dementia. This transdisciplinary project highlighted the challenges of participatory design in the context of dementia built environment. The importance of the topic was highlighted by clinicians and staff but there are still significant limitations in terms of research and methodologies. The workshops outcome was an inclusive code of conduct for participatory design and research for dementia patients, which will help to improve home and care environments for people with dementia. The framework involved aspects such as time, space, equipment in relation to people involved (carers, patients, proxies).

**Key messages:**

• The project created a framework to support dementia patients’ involvement for built environment decision making that considers aspects such as time, space and equipment to foster communication.

• The framework described the phases and the tools/methods in order to build trust and enable fluidity to accommodate dementia patients’ needs.

